# Vaccination Coverage Among Adolescents Aged 13–17 Years — National Immunization Survey-Teen, United States, 2024

**DOI:** 10.15585/mmwr.mm7430a1

**Published:** 2025-08-14

**Authors:** Cassandra Pingali, David Yankey, Laurie D. Elam-Evans, Adam Trahan, Lauri E. Markowitz, Carla L. DeSisto, Michelle Hughes, Madeleine R. Valier, Shannon Stokley, James A. Singleton

**Affiliations:** ^1^Immunization Services Division, National Center for Immunization and Respiratory Diseases, CDC; ^2^Military & Health Research Foundation, Laurel, Maryland; ^3^Division of Viral Diseases, National Center for Immunization and Respiratory Diseases, CDC; ^4^Division of Bacterial Diseases, National Center for Immunization and Respiratory Diseases, CDC.

**Summary**

**What is already known about this topic?**

Three vaccines are routinely recommended for adolescents: tetanus, diphtheria, and acellular pertussis vaccine (Tdap); quadrivalent meningococcal conjugate vaccine (MenACWY); and human papillomavirus (HPV) vaccine. On the basis of shared clinical decision-making, adolescents may also receive meningococcal B vaccine and should catch up on any missed childhood vaccines.

**What is added by this report?**

In this 2024 national study, coverage with ≥1 dose of Tdap, ≥1 dose of MenACWY, ≥1 dose of meningococcal B vaccine, ≥3 doses of hepatitis B vaccine, and ≥2 doses of measles, mumps, and rubella vaccine increased among adolescents aged 13–17 years compared with 2023. Coverage with ≥1 dose of HPV vaccine (78%) remains lower than ≥1-dose Tdap (91%) and ≥1-dose MenACWY (90%) coverage. HPV vaccination coverage did not increase for the third consecutive year; coverage continues to vary by metropolitan statistical area classification.

**What are the implications for public health practice?**

Health care providers can support adolescent health by discussing and strongly recommending Tdap, MenACWY, HPV vaccine, and other recommended vaccines and regularly reviewing patient records to check for recommended vaccines.

## Abstract

Three vaccines are recommended for routine administration to adolescents by the Advisory Committee on Immunization Practices: tetanus toxoid, reduced diphtheria toxoid, and acellular pertussis vaccine (Tdap); quadrivalent meningococcal conjugate vaccine (MenACWY); and human papillomavirus (HPV) vaccine. Data from the 2024 National Immunization Survey-Teen were analyzed to determine national, state, and selected local area vaccination coverage in 2024. Household response rate (21.0%) and receipt of adequate provider data for adolescents with completed interviews (42.8%) were comparable to prior survey years. Among 16,325 adolescents aged 13–17 years with adequate provider data included in the survey, coverage with ≥1 Tdap dose increased from 89.0% in 2023 to 91.3% in 2024; coverage with ≥1 MenACWY dose increased from 88.4% to 90.1%. HPV vaccination coverage remained stable for the third consecutive year; 78.2% of adolescents had received ≥1 dose, and 62.9% were up to date with the HPV vaccination series. Coverage with ≥1 Tdap dose was ≥90% in 39 states, with ≥1 MenACWY dose was ≥90% in 30 states, and with ≥1 dose of HPV vaccine was ≥80% in 26 states and the District of Columbia. Since 2016, lower HPV vaccination coverage in nonmetropolitan statistical areas (MSAs) compared with that in MSA principal cities has persisted, with an 11 percentage point difference in coverage with ≥1 HPV vaccine dose and percentage of adolescents up to date with HPV vaccination in 2024. Health care providers can support adolescent health by discussing and recommending vaccines, as well as reviewing patient records to ascertain whether adolescents are up to date with recommended vaccines.

## Introduction

Adolescent vaccination is an important tool for supporting health during adolescence and beyond. The Advisory Committee on Immunization Practices (ACIP) recommends that adolescents aged 11–12 years receive tetanus toxoid, reduced diphtheria toxoid, and acellular pertussis vaccine (Tdap); quadrivalent meningococcal conjugate vaccine (MenACWY); and human papillomavirus (HPV) vaccine. HPV vaccination may be started at age 9 years. A booster dose of MenACWY should be administered at age 16 years. On the basis of shared clinical decision-making, adolescents may receive a COVID-19 vaccine,* and persons aged 16–23 years may receive serogroup B meningococcal vaccine (MenB). In addition, adolescents should receive an annual influenza vaccination^†^ and catch up on any missed childhood vaccines (*1*). Since 2023, ACIP has recommended that pentavalent meningococcal vaccine (MenABCWY) can be used when both MenACWY and MenB are indicated at the same visit^§^ This report summarizes coverage with these vaccines in 2024 (excluding influenza and COVID-19 vaccines and MenABCWY vaccine, as limited data were available for MenABCWY vaccine in 2024) and compares coverage with that in 2023, using data from the National Immunization Survey-Teen (NIS-Teen).

## Methods

### Survey Methodology

NIS-Teen is an annual two-phase survey that monitors vaccination coverage in the United States among adolescents aged 13–17 years.^¶^ The first phase is a random-digit–dialed mobile telephone survey** of parents or guardians (parents) in households with eligible adolescents aged 13–17 years. The NIS-Teen mobile telephone sample is designed to meet target precision requirements using flagged telephone numbers from the NIS-Child sample and excludes telephone numbers in the National Immunization Survey do-not-call list (*2*). At the end of the household survey, permission is requested to contact the adolescent’s vaccination providers. The second phase of NIS-Teen is a mailed survey to the adolescent’s vaccination providers identified by the parent or guardian (parent) to obtain the adolescent’s vaccination history. NIS-Teen contacts all vaccination providers identified by the parent, including medical offices, health departments, pharmacies, and any other locations where the adolescent might have received vaccinations.

### Vaccination Coverage Estimates

Coverage estimates in this report were derived from provider-reported data on 16,325 adolescents aged 13–17 years^††^ who were born during January 2006–December 2011.^§§^ The household response rate^¶¶^ was 21.0%. Adequate provider data was received from providers for 42.8% of adolescents with completed interviews.*** These two rates reflect different types of response mechanisms. The household response rate measures participation by households that completed the household interviews, whereas the adequate provider data rate measures vaccination data availability among those who completed the household interviews. NIS-Teen uses a complex weighting process that includes adjustments for household nonresponse, provider nonresponse, and households without telephones. Weights are calibrated to known population totals by age, sex, race and ethnicity, and geography to improve representation (*2*). To address low response rates, nonresponse adjustments are incorporated in the weighting to reduce potential bias. In addition, statistical modeling techniques such as imputation and variance estimation methods are used to handle missing data and account for the complex survey design (*2*). Estimated vaccination coverage among adolescents aged 13–17 years in the 2024 survey year was compared with estimates from the 2023 survey year. Differences in vaccination coverage were determined using z-tests. p-values <0.05 were considered statistically significant. Analyses were conducted using SAS (version 9.4; SAS Institute) and SAS-callable SUDAAN (version 11; RTI International). This activity was reviewed by CDC, deemed not research, and was conducted consistent with applicable federal law and CDC policy.^†††^

## Results

### National and State Level Vaccination Coverage Among Adolescents Aged 13–17 Years

In 2024, among adolescents aged 13–17 years, ≥1-dose Tdap^§§§^ coverage increased 2.3 percentage points, to 91.3% from 89.0% in 2023, and ≥1-dose MenACWY^¶¶¶^ coverage increased 1.7 percentage points, to 90.1% from 88.4% in 2023 (Table 1) (Supplementary Figure). In 2024, 78.2% of adolescents aged 13–17 years had received ≥1 dose of HPV vaccine,**** and 62.9% were up to date with the HPV vaccination series,^††††^ similar to 2023 estimates (76.8% and 61.4%, respectively). Coverage with other recommended vaccines and catch-up vaccines^§§§§^ also increased: ≥1-dose MenB^¶¶¶¶^ coverage increased 4.5 percentage points among adolescents aged 17 years; ≥2-dose measles, mumps, and rubella (MMR) coverage and ≥3-dose hepatitis B (HepB) coverage both increased 1.3 percentage points among adolescents aged 13–17 years compared with coverage in 2023.

**TABLE 1:** Estimated vaccination coverage and varicella history among adolescents aged 13–17* years, by age at interview — National Immunization Survey-Teen, United States, 2024

Vaccine doses and varicella history	Age at interview, yrs, % (95% CI)					Total % (95% CI)	
	13 n = 3,143	14 n = 3,355	15 n = 3,294	16 n = 3,394	17 n = 3,139	2023 n = 16,568	2024 n = 16,325
**Tdap^†^ ≥1 dose**	89.4 (87.5–91.1)	92.1 (90.7–93.3)^§^	91.2 (89.6–92.6)	92.3 (90.7–93.6)^§^	91.4 (89.6–92.9)	**89.0 (87.9–90.0)**	**91.3 (90.6–92.0)^¶^**
MenACWY**							
≥1 dose	86.6 (84.3–88.5)	90.8 (89.2–92.3)^§^	89.8 (88.0–91.3)^§^	91.3 (89.6–92.7)^§^	91.9 (90.1–93.3)^§^	**88.4 (87.3–89.4)**	**90.1 (89.3–90.8)^¶^**
≥2 doses^††^	NA	NA	NA	NA	61.1 (58.3–63.8)	**59.7 (56.2–63.2)**	**61.1 (58.3–63.8)**
**HPV vaccine^§§^**							
All adolescents							
≥1 dose	71.1 (68.4–73.7)	77.0 (74.5–79.3)^§^	77.7 (75.3–80.0)^§^	82.9 (80.8–84.8)^§^	81.7 (79.5–83.7)^§^	**76.8 (75.4–78.1)**	**78.2 (77.2–79.2)**
Up to date^¶¶^	50.5 (47.5–53.4)	60.6 (57.7–63.4)^§^	64.6 (61.7–67.3)^§^	69.6 (66.9–72.2)^§^	68.1 (65.4–70.7)^§^	**61.4 (59.9–63.0)**	**62.9 (61.6–64.1)**
Adolescent girls							
≥1 dose	73.1 (69.2–76.6)	76.8 (73.0–80.1)	77.6 (74.1–80.8)	84.9 (82.1–87.4)^§^	82.4 (79.0–85.3)^§^	**78.5 (76.7–80.2)**	**79.1 (77.6–80.5)**
Up to date	51.4 (47.2–55.5)	62.0 (57.9–65.9)^§^	66.0 (62.0–69.7)^§^	70.8 (66.6–74.7)^§^	70.1 (66.2–73.8)^§^	**64.0 (61.9–66.1)**	**64.3 (62.5–66.1)**
Adolescent boys							
≥1 dose	69.3 (65.4–73.0)	77.3 (73.9–80.3)	77.8 (74.4–80.9)	81.0 (78.0–83.8)	81.1 (78.0–83.8)	**75.1 (73.0–77.1)**	**77.4 (75.9–78.8)**
Up to date	49.6 (45.5–53.8)	59.1 (55.0–63.1)	63.2 (59.0–67.2)	68.6 (64.9–72.0)	66.2 (62.4–69.7)	**59.0 (56.7–61.2)**	**61.6 (59.8–63.3)**
MenB***							
≥1 dose	NA	NA	NA	NA	36.9 (34.1–39.7)	**32.4 (29.3–35.6)**	**36.9 (34.1–39.7)^¶^**
≥2 doses	NA	NA	NA	NA	15.9 (13.8–18.0)	**12.8 (10.7–15.3)**	**15.9 (13.8–18.0)**
**MMR ≥2 doses**	93.3 (91.7–94.7)	92.8 (90.8–94.3)	92.9 (91.5–94.1)	92.3 (90.7–93.6)	91.6 (89.8–93.1)	**91.3 (90.2–92.3)**	**92.6 (91.9–93.2)^¶^**
**Hep A vaccine ≥2 doses^†††^**	88.6 (86.5–90.5)	88.6 (86.6–90.4)	86.4 (84.0–88.5)	86.6 (84.3–88.6)	85.2 (83.0–87.1)^§^	**86.9 (85.7–88.0)**	**87.1 (86.1–88.0)**
**Hep B vaccine ≥3 doses**	93.1 (91.5–94.5)	93.3 (91.7–94.6)	91.6 (89.5–93.3)	92.3 (90.8–93.6)	90.8 (88.8–92.4)	**90.9 (89.8–91.9)**	**92.2 (91.5–92.9)^¶^**
**History of varicella disease^§§§^**	6.5 (5.2–8.1)	5.5 (4.6–6.7)	7.2 (5.9–8.8)	9.2 (7.4–11.2)^§^	8.7 (7.2–10.5)	**7.3 (6.4–8.2)**	**7.5 (6.8–8.2)**
**No history of varicella disease**							
≥1 dose varicella vaccine	96.4 (95.1–97.3)	96.2 (95.0–97.1)	95.4 (94.2–96.3)	95.1 (93.7–96.2)	93.2 (91.3–94.7)^§^	**94.6 (93.8–95.4)**	**95.3 (94.7–95.8)**
≥2 doses varicella vaccine	92.9 (91.3–94.3)	92.3 (90.3–93.9)	91.3 (89.7–92.7)	92.2 (90.5–93.6)	90.8 (88.8–92.5)	**90.8 (89.8–91.8)**	**91.9 (91.1–92.6)**
**History of varicella disease or receipt of ≥2 doses varicella vaccine**	93.4 (91.8–94.7)	92.7 (90.9–94.2)	91.9 (90.4–93.2)	92.9 (91.3–94.2)	91.6 (89.8–93.1)	**91.5 (90.5–92.4)**	**92.5 (91.8–93.2)**

**Abbreviations:** Hep A = hepatitis A; Hep B = hepatitis B; HPV = human papillomavirus; MenACWY = quadrivalent meningococcal conjugate vaccine; MenB = serogroup B meningococcal vaccine; MMR = measles, mumps, and rubella vaccine; NA = not applicable; NIS-Teen = National Immunization Survey-Teen; Tdap = tetanus toxoid, reduced diphtheria toxoid, and acellular pertussis vaccine.

* Adolescents (16,325) in the 2024 NIS-Teen were born during January 2006–December 2011.

**^†^** Includes percentages receiving Tdap among persons aged ≥10 years.

^§^ Statistically significant difference (p<0.05) in estimated vaccination coverage by age: referent group was adolescents aged 13 years.

^¶^ Statistically significant difference (p<0.05) compared with 2023 NIS-Teen estimates.

** Includes percentages receiving MenACWY or meningococcal vaccine of unknown type.

^††^ ≥2 doses of MenACWY or meningococcal vaccine of unknown type. Calculated only among adolescents who were aged 17 years at interview. Does not include adolescents who received their first dose of MenACWY at age ≥16 years.

^§§^ Nine-valent, quadrivalent, or bivalent HPV vaccine. For ≥1 dose and up-to-date HPV vaccination measures, percentages are reported among adolescent girls and adolescent boys combined (16,325), for adolescent girls only (7,707), and for adolescent boys only (8,618).

^¶¶^ Up-to-date HPV vaccination status includes receipt of ≥3 HPV vaccine doses, or receipt of 2 doses when the first dose was initiated at age <15 years, and ≥5 months minus 4 days had elapsed between the first and second doses (General Best Practices for Immunization | Vaccines & Immunizations | CDC). This update to the HPV vaccination recommendation occurred in December 2016. Some adolescents might have received more than the 2 or 3 recommended HPV vaccine doses.

*** Calculated among adolescents who were aged 17 years at the time of interview with vaccine administered based on shared clinical decision-making; ≥2 doses of MenB required correct interval between first and second dose (Meningococcal Vaccination: Recommendations of the Advisory Committee on Immunization Practices, United States, 2020 | MMWR) and excluded those with unknown type of meningococcal vaccine. Although a pentavalent meningococcal vaccine was available in 2024, this analysis excluded this vaccine due to limited data availability.

^†††^ In July 2020, the Advisory Committee on Immunization Practices revised recommendations for Hep A vaccination to include catch-up vaccination for children and adolescents aged 2–18 years who have not previously received Hep A vaccine at any age.

^§§§^ By parent or guardian report or provider records.

Vaccination coverage varied by jurisdiction. The largest variation in coverage by jurisdiction was among adolescents up to date with HPV vaccination, which ranged from 39.1% in Mississippi to 79.8% in Massachusetts (Supplementary Table). In 2024, coverage of ≥90% among adolescents aged 13–17 years was observed for ≥1 dose of Tdap in 39 states and ≥1 dose of MenACWY in 30 states. Coverage with ≥1 dose of HPV vaccine was ≥80% in 26 states and the District of Columbia (DC), and at least 65% of adolescents were up to date with HPV vaccination in 26 states and DC. Compared with 2023, coverage with at least one vaccine routinely recommended for adolescents increased in Florida, Georgia, Kentucky, and Virginia among adolescents aged 13–17 years in 2024.*****

### Vaccination Coverage Among Adolescents Aged 13–17 Years, by Metropolitan Statistical Area**^†††††^**

In 2024, among adolescents aged 13–17 years, coverage with ≥1 and ≥2 doses of MenACWY was 3.6 and 10.0 percentage points lower, respectively, among those living in nonmetropolitan statistical areas (MSAs) (mostly rural areas) compared with those living in MSA principal cities (mostly urban areas) (Table 2). In addition, coverage with ≥1 dose of HPV vaccine, and the percentage of adolescents who were up to date with HPV vaccination were 4.7 and 3.0 percentage points lower, respectively, in MSA nonprincipal cities (mostly suburban areas) and 10.5 and 10.8 percentage points lower, respectively, in mostly rural areas compared with coverage in mostly urban areas. The gap in HPV vaccination coverage among adolescents aged 13–17 years living in mostly rural areas and those living in mostly urban areas has changed little during the previous 9 years, and the magnitude of the difference remains largely unchanged since 2016 (Figure). Coverage with ≥1 dose of Tdap was similar across the three MSA categories (Table 2).

**TABLE 2:** Estimated vaccination coverage and receipt of a provider recommendation for human papillomavirus* vaccine among adolescents aged 13–17^†^ years, by metropolitan statistical area status^§^ — National Immunization Survey-Teen, United States, 2024

Vaccine or receipt of provider recommendation	MSA status, % (95% CI)		
	Non-MSA (mostly rural) n = 3,033	MSA nonprincipal city (mostly suburban) n = 6,685	MSA principal city (mostly urban) (Ref) n = 6,607
**Tdap^¶^ ≥1 dose**	90.0 (88.2–91.8)	91.2 (90.2–92.2)	91.8 (90.8–92.9)
MenACWY**			
≥1 dose	86.9 (85.0–88.9)^††^	90.6 (89.5–91.7)	90.5 (89.2–91.7)
≥2 doses^§§^	52.5 (46.5–58.4)^††^	62.2 (58.2–66.2)	62.5 (58.1–66.9)
**HPV^¶¶^ vaccine**			
≥1 dose	71.2 (68.6–73.8)^††^	77.0 (75.4–78.5)^††^	81.7 (80.1–83.3)
Up to date***	54.8 (52.0–57.6)^††^	62.6 (60.8–64.5)^††^	65.6 (63.5–67.6)
**Received a provider recommendation for HPV vaccine**	65.6 (62.9–68.3)^††^	70.9 (69.0–72.7)	69.4 (67.3–71.3)
**HPV vaccine (with a provider recommendation) (Ref)**			
≥1 dose	77.2 (74.0–80.4)^†††^	82.3 (80.7–83.9)^†††^	86.0 (84.4–87.6)
Up to date	61.1 (57.6–64.5)^†††^	68.4 (66.4–70.4)^†††^	71.7 (69.5–74.0)
**HPV vaccine (without a provider recommendation)**			
≥1 dose	57.2 (51.9–62.4)^†††,§§§^	58.7 (54.4–63.1)^†††,§§§^	68.4 (63.8–72.9)^§§§^
Up to date	39.5 (33.9–45.2)^§§§^	43.2 (38.8–47.5)^§§§^	45.7 (40.7–50.7)^§§§^

**Abbreviations:** HPV = human papillomavirus; MenACWY = quadrivalent meningococcal conjugate vaccine; MSA = metropolitan statistical area; NIS-Teen = National Immunization Survey-Teen; Ref = referent group; Tdap = tetanus toxoid, reduced diphtheria toxoid, and acellular pertussis vaccine.

* Parents or guardians were asked, “Has a doctor or other health care professional ever recommended that [teen name] receive HPV shots?” A similar question was not asked of parents and guardians regarding Tdap and MenACWY vaccines in 2024.

^†^ Adolescents (16,325) in the 2024 NIS-Teen were born during January 2006–December 2011. Coverage estimates in this table were calculated using only SAS (version 9.4; SAS Institute) survey procedures.

^§^ MSA status was determined from household reported city and county of residence and was grouped into three categories: MSA principal city, MSA nonprincipal city, and non-MSA. Non-MSAs include urban populations not located within an MSA and completely rural areas. Metropolitan and Micropolitan | United States Census Bureau

^¶^ Includes percentages of adolescents aged ≥10 years receiving Tdap.

** Includes percentages of adolescents receiving MenACWY and meningococcal-unknown type vaccine.

^††^ Statistically significant difference (p<0.05) by MSA; Ref was adolescents living in MSA principal city areas.

^§§^ ≥2 doses of MenACWY or meningococcal-unknown type vaccine. Calculated only among adolescents who were aged 17 years at interview. Does not include adolescents who received their first dose of MenACWY at age ≥16 years.

^¶¶^ Nine-valent, quadrivalent, or bivalent HPV vaccine. For ≥1 dose and up-to-date HPV vaccination measures, percentages are reported among adolescent girls and boys combined (16,325) and for adolescent girls only (7,707) and adolescent boys only (8,618).

*** Up-to-date HPV vaccination status includes receipt of ≥3 doses or receipt of 2 doses when the first HPV vaccine dose was initiated at age <15 years, and ≥5 months minus 4 days had elapsed between the first and second doses (General Best Practices for Immunization | Vaccines & Immunizations | CDC). This update to the HPV vaccination recommendation occurred in December 2016. Some adolescents might have received more than the 2 or 3 recommended HPV vaccine doses.

^†††^ Statistically significant difference (p<0.05) in estimated HPV vaccination coverage by provider recommendation for HPV vaccine across MSA status; Ref was adolescents living in an MSA principal city.

^§§§^ Statistically significant difference (p<0.05) in estimated HPV vaccination coverage by provider recommendation for HPV vaccine within MSA status; Ref was adolescents who received an HPV vaccine recommendation from a provider.

**FIGURE:**
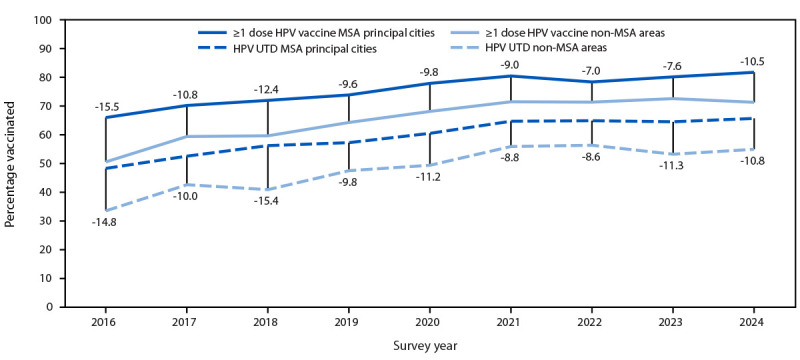
Difference in vaccination coverage with ≥1 dose of human papillomavirus vaccine* and percentage of adolescents up to date with human papillomavirus vaccination^†^ among adolescents aged 13–17 years, by metropolitan statistical area status^§^ — National Immunization Survey-Teen, United States, 2016–2024 **Abbreviations**: HPV = human papillomavirus; MSA = metropolitan statistical area; UTD = up to date. * The difference in ≥1-dose HPV vaccination coverage by survey year between adolescents living in MSA principal cities and non-MSA areas. ^†^ The difference in percentage of adolescents UTD with HPV vaccination by survey year among adolescents living in MSA principal cities and non-MSA areas. ^§^ MSA status was determined from household reported city and county of residence and was grouped into three categories: MSA principal city, MSA nonprincipal city, and non-MSA. Non-MSAs include urban populations not located within an MSA and completely rural areas. "https://www.census.gov/programs-surveys/metro-micro.html"Metropolitan and Micropolitan | U.S. Census Bureau

### HPV Vaccination Coverage by MSA Status and Receipt of a Provider Recommendation for HPV Vaccine

In 2024, the parents of 69.4% of adolescents living in mostly urban areas, 70.9% of those living in mostly suburban areas, and 65.6% of those living in mostly rural areas received a provider recommendation (self-reported by parent) for their adolescent to receive HPV vaccine. In all MSA areas, ≥1-dose HPV vaccination coverage was lower among adolescents who did not receive an HPV vaccination recommendation from a provider (range = 57.2%–68.4%) than among those who did receive a provider recommendation (range = 77.2%–86.0%). Among adolescents who received a provider HPV vaccination recommendation, ≥1-dose HPV vaccination coverage was 8.8 percentage points lower in mostly rural areas and 3.7 percentage points lower in mostly suburban areas than in mostly urban areas; similarly, the percentage of adolescents who were up to date with HPV vaccination was 10.6 and 3.3 percentage points lower in mostly rural and mostly suburban areas, respectively, than in mostly urban areas. Similarly, among adolescents whose parent did not receive a provider recommendation for HPV vaccine, ≥1-dose HPV vaccination coverage among those in mostly rural and mostly suburban areas was 11.2 and 9.7 percentage points, respectively, lower than coverage among those living in mostly urban areas.

## Discussion

Among adolescents aged 13–17 years, coverage with ≥1 Tdap dose, ≥1 MenACWY dose, ≥1 MenB dose (assessed among adolescents aged 17 years), ≥2 MMR doses, and ≥3 HepB doses increased in 2024; ≥1-dose Tdap coverage and ≥1-dose MenACWY coverage was ≥90% in a majority of states. These findings highlight progress in public health activities to improve vaccination coverage (*3*,*4*).

HPV vaccination coverage has not changed among adolescents aged 13–17 years for 3 consecutive years; for the previous 9 years, HPV vaccination coverage has remained lower among adolescents living in mostly rural areas compared with coverage among those in mostly urban areas. Additional activities are needed to improve HPV vaccination coverage among adolescents.

A strong provider recommendation is associated with increased likelihood of being vaccinated (*5*); however, in this analysis, adolescents living in mostly rural areas were less likely to receive an HPV vaccination recommendation from a provider than were those living in mostly urban areas. In 2024, ≥1-dose HPV coverage and the percentage of adolescents up to date with HPV vaccination were consistently higher among those who received a provider recommendation for vaccination. These findings highlight the influence of provider recommendations and the potential for strong provider recommendations to improve vaccination coverage. Ongoing conversations with families can emphasize the role of the HPV vaccine in cancer prevention and the importance of other vaccines recommended for adolescents.

Although these findings are consistent with previous research examining HPV vaccination coverage by MSA status, reasons for these differences are not well understood (*6*,*7*). Even when rural and suburban families received a provider recommendation for HPV vaccination, their adolescent children were less likely to be vaccinated than were those living in mostly urban areas. This might indicate additional barriers to vaccination such as transportation challenges, fewer opportunities for well-child visits, concerns about vaccine safety, or differing attitudes and beliefs that influence vaccine acceptance. A better understanding of these barriers is needed to guide development of strategies that support state programs, health departments, and providers to strengthen outreach and education and ensure that all adolescents, regardless of geographic location, receive information about and access to HPV vaccine and other recommended vaccines.

### Limitations

The findings in this report are subject to at least two limitations. First, the household response rate was low, and only 42.8% of those who completed interviews had adequate provider data. Selection bias might exist if the respondents in the survey differ systematically from nonrespondents, and these differences are not accounted for by survey weighting. Although NIS-Teen applies weighting adjustments to reduce bias, direct assessment of systematic differences between respondents and nonrespondents is limited because of lack of data on nonrespondents. Second, although estimates are adjusted for household and provider nonresponse and households without a telephone, bias in the estimates might remain, which might result in overestimations or underestimations of coverage. Each year a total survey error (TSE) assessment is created in conjunction with release of NIS-Teen data. The 2023 TSE assessment indicated that NIS-Teen estimates might underestimate actual coverage, with the largest underestimation occurring for up-to-date HPV vaccination status (−5.2 percentage points), primarily attributed to incomplete ascertainment of vaccination status (e.g., if parents did not report all vaccination providers or if the provider either did not respond to the provider survey or did not report all vaccines received by the adolescent) (*8*). The 2024 TSE estimates were similar to those from previous years for the vaccines assessed (NORC at the University of Chicago, CDC, unpublished data, 2025).

### Implications for Public Health Practice

Health care providers can improve the health and safety of adolescents and their communities through continued education and engagement with families about the importance of vaccines and their role in supporting adolescent health. Health care providers can also routinely review adolescent patients’ immunization records to ascertain whether they are up to date with recommended vaccines. State, local, and territorial health departments can further use NIS-Teen findings by evaluating local vaccination data sources, such as immunization information system data, to identify geographic areas with low coverage to gain a more comprehensive understanding of vaccination coverage in their jurisdiction (*9*,*10*). Using these insights, health departments can work with health care providers and communities to improve local vaccine access and increase adolescent vaccination coverage.

## Footnotes

* COVID-19 vaccination is recommended on the basis of shared clinical decision-making for all persons aged 6 months–17 years (Staying Up to Date with COVID-19 Vaccines | COVID-19 | CDC). Estimates of COVID-19 vaccination coverage are available at COVID-19 Vaccination Coverage and Vaccine Confidence Among Children | COVIDVaxView | CDC.

† Influenza vaccination is recommended for all persons aged ≥6 months. Influenza vaccination coverage estimates are available at FluVaxView | FluVaxView | CDC.

§ On October 25, 2023, the ACIP recommended that a pentavalent MenABCWY vaccine (MenACWY-TT/MenB-FHbp) may be administered to persons aged ≥10 years when both a quadrivalent meningococcal conjugate vaccine (MenACWY) and meningococcal B vaccine (MenB) are indicated at the same visit (Use of the Pfizer Pentavalent Meningococcal Vaccine Among Persons Aged ≥10 Years: Recommendations of the Advisory Committee on Immunization Practices ― United States, 2023 | MMWR ). On April 16, 2025, ACIP recommended an additional pentavalent MenABCWY vaccine (MenACWY-CRM/MenB-4C) (PENMENVY | FDA ) could be used when both MenACWY and MenB are indicated at the same visit. More information on MenABCWY is available at Meningococcal Vaccine Recommendations | Meningococcal | CDC.

¶ Local areas that received federal immunization funds under Section 317 of the Public Health Service Act were sampled separately. Those areas included Bexar County, Texas; Chicago, Illinois; Houston, Texas; New York, New York; and Philadelphia County, Pennsylvania. Two U.S. territories, Guam and Puerto Rico, were sampled separately in 2024.

** Persons living in all identified households with mobile telephones were eligible for interview. Sampling weights were adjusted for survey nonresponse, adolescent multiplicity (number of chances for selection), and noncoverage of the survey sampling frame, and were calibrated to known population totals. During 2011–2018, NIS-Teen sampled from a landline frame in addition to a mobile telephone frame; therefore, sampling weights were also adjusted for overlapping samples of mixed telephone users. A description of NIS-Teen single-frame survey methodology and its effect on reported vaccination estimates is available at National Immunization Survey-Teen, 2016-2017 | TeenVaxView | CDC.

†† The 2024 NIS-Teen sample included 7,707 adolescent girls and 8,618 adolescent boys. Adolescents from Guam (106) and Puerto Rico (234) were excluded from the national estimates.

§§ Estimates in this report include persons who might have received vaccinations on time or as catch-up.

¶¶ The Council of American Survey Research Organizations household response rate is the product of three other rates: 1) the resolution rate (the proportion of telephone numbers that can be identified as either business or residence), 2) the screening rate (the proportion of qualified households that complete the screening process), and 3) the cooperation rate (the proportion of contacted eligible households for which a completed interview is obtained).

*** Adolescents with receipt of one or more non–COVID-19 vaccine doses reported by a provider and those who had received no vaccinations were considered to have adequate provider data. “No vaccinations” indicates that the vaccination status is known because the parent or guardian indicated there were no vaccinations, and the provider returned no immunization history forms or returned them indicating that no vaccinations had been administered.

††† 45 C.F.R. part 46.102(l)(2), 21 C.F.R. part 56; 42 U.S.C. Sect. 241(d); 5 U.S.C. Sect. 552a; 44 U.S.C. Sect. 3501 et seq.

§§§ Tdap vaccination coverage represents coverage with ≥1 Tdap dose at age ≥10 years.

¶¶¶ Meningococcal conjugate vaccination coverage represents coverage with ≥1 MenACWY dose or meningococcal (unknown type) vaccine.

**** HPV vaccination coverage includes receipt of any HPV vaccine and does not distinguish among nine-valent, quadrivalent, or bivalent vaccines. Some adolescents might have received more than the 2 or 3 recommended HPV vaccine doses.

†††† Up-to-date HPV vaccination status includes receipt of ≥3 doses or receipt of 2 doses when the first HPV vaccine dose was initiated at age <15 years, and ≥5 months minus 4 days had elapsed between receipt of the first and second doses (General Best Practices for Immunization | Vaccines & Immunizations | CDC). This update to the HPV vaccination recommendation occurred in December 2016. Some adolescents might have received more than the 2 or 3 recommended HPV vaccine doses.

§§§§ Vaccinations against hepatitis A; hepatitis B; varicella; and measles, mumps, and rubella are considered childhood vaccines and are recommended for adolescents who are not up to date with these vaccinations. Except as noted, coverage estimates for ≥1 and ≥2 varicella vaccine doses were obtained among adolescents with no history of varicella disease.

¶¶¶¶ MenB vaccination is not routinely recommended for all adolescents. Vaccines are administered to adolescents and young adults aged 16–23 years based on shared clinical decision-making. Coverage estimates for ≥1 and ≥2 MenB doses were calculated among adolescents who were aged 17 years at the time of interview; ≥2 doses of MenB required the correct interval between the first and second dose and excluded persons who had received an unknown type of meningococcal vaccine.

***** In 2024, coverage with ≥1 Tdap dose increased in Florida and Georgia; coverage with ≥1 MenACWY dose increased in Florida, Georgia, and Virginia; coverage with ≥1 HPV vaccine dose increased in Florida; and the percentage of adolescents who were up to date with HPV vaccination increased in Georgia and Kentucky.

††††† MSA status was determined from household-reported city and county of residence and was grouped into three categories: MSA principal city, MSA nonprincipal city, and non-MSA. Non-MSAs include urban populations not located within an MSA and completely rural areas. Metropolitan and Micropolitan | United States Census Bureau
